# Structural basis of ligand activation and inhibition in a mammalian TRPV4 ion channel

**DOI:** 10.1038/s41421-023-00579-3

**Published:** 2023-07-10

**Authors:** Wenxuan Zhen, Zhijun Zhao, Shenghai Chang, Xiaoying Chen, Yangzhuoqun Wan, Fan Yang

**Affiliations:** 1 Department of Biophysics and Kidney Disease Center of the First Affiliated Hospital, Zhejiang University School of Medicine, Hangzhou, Zhejiang China; 2 Liangzhu Laboratory, Zhejiang University Medical Center, Hangzhou, Zhejiang China; 3 Alibaba-Zhejiang University Joint Research Center of Future Digital Healthcare, Hangzhou, Zhejiang, China; 4 Department of Pathology of Sir Run Run Shaw Hospital, Zhejiang University School of Medicine, Hangzhou, Zhejiang China; 5 Center of Cryo Electron Microscopy, Zhejiang University, Hangzhou, Zhejiang China

**Keywords:** Cryoelectron microscopy, Protein folding

Dear Editor,

The transient receptor potential vanilloid 4 (TRPV4) channel is a polymodal receptor that is activated by warm temperature, osmolarity changes, and many ligands^1^. Like other members in the TRPV family, TRPV4 is critically involved in a plethora of physiological processes like temperature sensation, osmoregulation, and mechanotransduction. Importantly, TRPV4 has the largest number of mutations associated with human diseases^2^ among the TRPV channels. Therefore, TRPV4 has been heavily targeted in drug developments^3^ against diseases like chronic cough (NCT03372603) and heart failure (NCT02497937 and NCT02119260). Despite the high-resolution structures of all other members in the TRPV family in mammals have been resolved^4^, to date the only peer-reviewed and published structure of TRPV4 is from *Xenopus tropicalis* (xTRPV4), which is in the apo state with an S4-S5 linker distinct from most of TRPV channels^5^, offering limited information regarding the ligand gating mechanism of this channel.

To understand how the TRPV4 channel is activated and inhibited by ligands and to facilitate drug development targeting this channel in the future, we have resolved the structures of mouse TRPV4 in the apo state (mTRPV4_apo_, 3.6 Å), in complex with the agonist GSK1016790A (GSK101) (mTRPV4_GSK101_, 3.6 Å), in complex with both GSK101 and ruthenium red (RR) (mTRPV4_GSK101_RR_, 3.7 Å) and in complex with Agonist-1 and RR (mTRPV4_Agonist1_RR_, 3.9 Å) states by cryo-electron microscopy (cryo-EM) (Supplementary Table S1). We first observed that mTRPV4_apo_ is a homotetramer (Fig. 1a, b; Supplementary Fig. S1), where the S1 to S4 bundle and pore domain composed of S5 and S6 are organized in a domain-swapped manner (Fig. 1c). The mTRPV4_apo_ is in a closed state, with the residue M718 forming the permeation-restricting site in S6 (Fig. 1d). Compared to xTRPV4 in the apo state (PDB ID: 6BBJ), we find that the S4-S5 linker in mouse TRPV4 adopts an alpha-helical conformation similar to that in TRPV1, TRPV2, or TRPV3, but not the loop as in xTRPV4 (Fig. 1c, indicated with an arrow; Supplementary Fig. S2). Moreover, though the subunit in both xTRPV4 and mTRPV4 is domain-swapped, the pore domain in xTRPV4 locates much closer to the S1 to S4 bundle as compared to those in mTRPV4 (Fig. 1e, subunits in green and tan, respectively). Therefore, we believe that the structure of xTRPV4 is less relevant when the function of TRPV4 is considered in mammals.

**Fig. 1:**
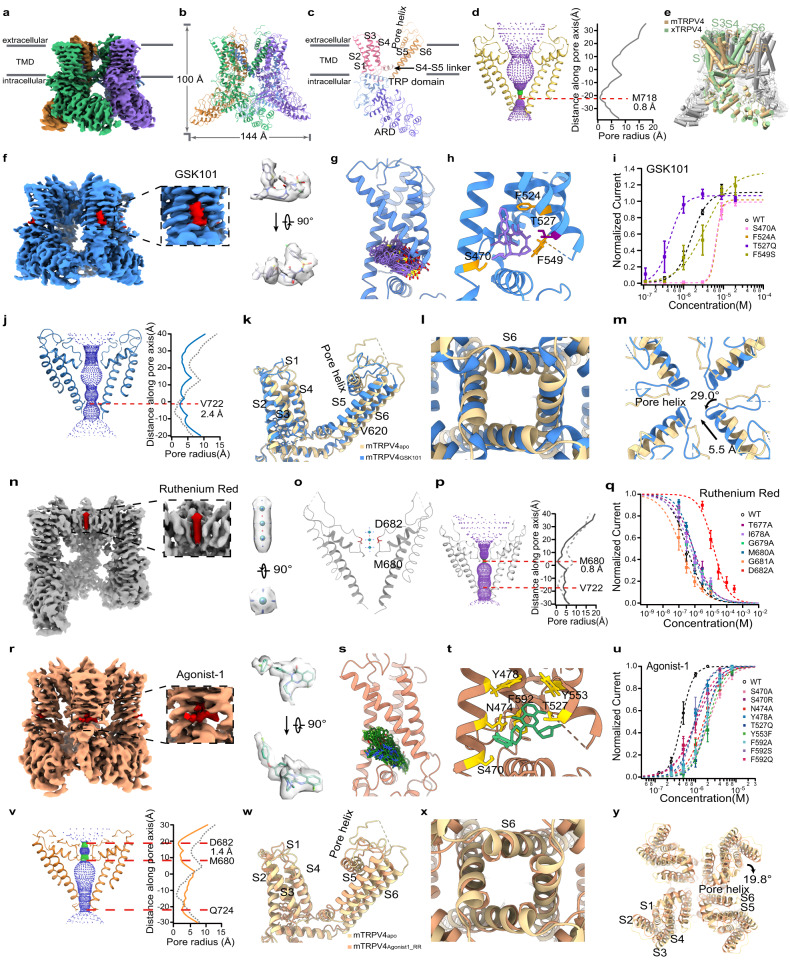
Structures of mouse TRPV4 in the apo and ligand bound states. **a** 3D reconstruction of mTRPV4_apo_ with each subunit individually colored. The gray bars define the position of the cell membrane. **b** Cartoon representation of mTRPV4_apo_ with each subunit individually colored. **c** Cartoon representation of one subunit of mTRPV4_apo_ with domains colored in a rainbow. **d** Profile of pore radii in mTRPV4_apo_. The region in red is too narrow to allow a water molecule to pass. **e** Cylinder representation of mTRPV4 compared to xTRPV4 in the apo state. **f** 3D reconstruction of mTRPV4_GSK101_.The electron density of a GSK101 molecule is highlighted in red. **g** Ensemble plot of the GSK101 molecule in the cavity formed between S1 to S4 of mTRPV4 in all-atom MD simulations. **h** GSK101 inside mTRPV4, with the labeling of key residues in the binding sites. **i** Concentration-response curves of GSK101 activation in WT mTRPV4 and mutants (*n* = 3–8 cells). Data were presented as mean ± s.e.m. **j** Profile of pore radii in mTRPV4_GSK101_ compared with mTRPV4_apo_. **k** Side view of conformational changes of mTRPV4_GSK101_ compared with mTRPV4_apo_. Only one subunit was shown for clarity. **l** Bottom view from the intracellular side of conformational changes of mTRPV4_GSK101_ compared with mTRPV4_apo_. **m** Top view from the extracellular side of conformational changes of mTRPV4_GSK101_ compared with mTRPV4_apo_. **n** 3D reconstruction of mTRPV4_GSK101_RR_. The electron density of a Ruthenium Red molecule is highlighted in red. **o** Cartoon representation of Ruthenium Red in mTRPV4. Key residues close to Ruthenium Red are shown. **p** Profile of pore radii in mTRPV4_GSK101_RR_. **q** Concentration-response curves of Ruthenium Red inhibition in WT TRPV4 and mutants (*n* = 3–4 cells). Data were presented as mean ± s.e.m. **r** The electron density of an Agonist-1 molecule is highlighted in red. **s** Ensemble plot of the Agonist-1 molecule in the binding pocket formed between S1 to S4 of mTRPV4 in all-atom MD simulations. **t** Cartoon representation of Agonist-1 in mTRPV4. Key residues close to Ruthenium Red are shown. **u** Concentration-response curves of Agonist-1 activation in WT TRPV4 and mutants (*n* = 3–4 cells). Data were presented as mean ± s.e.m. **v** Profile of pore radii in mTRPV4_Agonist1_RR_. **w** Side view of the conformational changes of mTRPV4_Agonist1_RR_ compared with mTRPV4_apo_. Only one subunit was shown for clarity. **x** Bottom view from the intracellular side of mTRPV4_Agonist1_RR_ compared with mTRPV4_apo_. **y** Top view from the extracellular side of the conformational changes of mTRPV4_Agonist1_RR_ compared with mTRPV4_apo_.

To investigate how small molecules such as GSK101^6^ activate TRPV4, we resolved the structure of the GSK101-mTRPV4 complex (mTRPV4_GSK101_) and observed that GSK101 bound to the cytosol-facing cavity formed between S1 to S4 (Fig. 1f, density in red; Supplementary Fig. S3). We then performed an all-atom molecular dynamic simulation of mTRPV4_GSK101_ for 200 ns. With the ensemble plot of GSK101 (Fig. 1g), we observed that this molecule is stably bound within the cavity. Such a binding configuration of GSK101 predicated that residues like S470, F524, and F549 would interact with this molecule (Fig. 1h).

To functionally validate the GSK101-binding site, we mutated these residues and performed whole-cell patch-clamp recordings. To find a reference agonist that does not bind to the cytosol-facing cavity formed between S1 to S4, we first tried to determine the structure of mTRPV4 in complex with RN-1747^7^. After adding 100 μM RN-1747 to the mTRPV4 protein sample and resolving the density map with cryo-EM (Supplementary Fig. S4), we did not observe any density resembling RN-1747 in the S1-S4 pocket (Supplementary Fig. S5a). Furthermore, when we mutated the residues in the S1-S4 pocket, which lead to large changes in GSK101 or Agonist-1 activation (Fig. 1i, u), the concentration-response curves of RN-1747 remained unchanged (Supplementary Fig. S5b and Table S5). Therefore, though we have not directly observed where RN-1747 binds to in mTRPV4, we believe that RN-1747 does not bind to the S1-S4 pocket employed by GSK101 or Agonist-1. Then by using RN-1747 as the reference agonist (Supplementary Fig. S5c), we observed that the GSK101 concentration–response curves of mutants were largely shifted to higher concentration (Fig. 1i; Supplementary Table S2). Interestingly, in the T527Q mutant, the concentration–response curve was left-shifted so that GSK101 activated this mutant at lower concentrations.

The mTRPV4_GSK101_ is in an open state as suggested by its pore radius profile (Fig. 1j). By comparing mTRPV4_GSK101_ with mTRPV4_apo_, we observed that GSK101 induced an upward movement of the S4-S5 linker by about 5 Å measured at the CA atoms at V620 (Fig. 1k), which further caused the large lift in S5 and expansion of S6 bundle crossing, leading to the opening the channel (Fig. 1l). Moreover, while the intracellular domains remained largely unchanged (Supplementary Fig. S6a, b), GSK101 also induced conformational changes in the pore region, causing the pore helix to rotate counter-clockwise by about 29° when viewed from the extracellular side and move towards pore center by 5.5 Å measured at CA atoms at F669 (Fig. 1m).

RR is a commonly used inhibitor of many TRP channels, but to date, only the structure of TRPV6 in complex with RR has been resolved^8^. RR binds inside the selectivity filter of TRPV6 to block ion permeation in this channel. To understand how RR blocks the TRPV4 channel, we determined the structure of the GSK101-RR-mTRPV4 complex (mTRPV4_GSK101_RR_). In this complex, GSK101 also binds to the same cavity with a similar configuration as in mTRPV4_GSK101_ (Supplementary Fig. S7). We further observed a rod-like density in the extracellular entrance of the selectivity filter in TRPV4 (Fig. 1n, density in red, the subunit in front was omitted for clarity), which has been absent in mTRPV4_apo_ and mTRPV4_GSK101_ and fits well with the chemical structure of RR molecule. In mTRPV4_GSK101_RR_, RR interacted with residues lining the selectivity filter (Fig. 1o). While the S6 bundle crossing in mTRPV4_GSK101_RR_ remains to be open, binding of RR induced constriction in pore radius near the selectivity filter at residue M680 (Fig. 1p). By performing alanine scan in the selectivity filter (Fig. 1q), we observed that while these mutants were still functional as they were activated by 2 µM GSK101 (Supplementary Fig. S7), D682A mutant exhibited much reduced RR inhibition with a large right-shift in a concentration-response curve in patch-clamp recordings (IC50 of D682A and WT: 15.6 ± 3.2 µM and 0.3 ± 0.1 µM, respectively, Supplementary Table S3). Therefore, RR directly blocked the entrance of the pore and narrows the selectivity filter of TRPV4 to inhibit the channel.

To further consolidate the ligand activation and inhibition mechanisms in TRPV4, we resolved TRPV4 in complex with RR and Agonist-1 (mTRPV4_Agonist1_RR_) (Supplementary Fig. S8), which is another activator of TRPV4^9^. Interestingly, we observed that Agonist-1 also binds to the GSK101-binding pocket in the S1 to S4 bundle (Fig. 1r). With the all-atom molecular dynamic simulation of mTRPV4_Agonist1_RR_ for 200 ns, we observed that Agonist-1 stably bound within the cavity (Fig. 1s). Such a binding configuration of Agonist-1 predicated that residues like F592, Y553, and N474 would be in proximity with this molecule (Fig. 1t). We then mutated these residues and with patch-clamp recordings, we observed that concentration–response curves of the mutants were all shifted to higher concentration (Fig. 1u; Supplementary Table S4), which is consistent with our cryo-EM observations. Though we did not observe any electron density resembling RR in the pore of mTRPV4_Agonist1_RR_, which is likely due to the incomplete inhibition of Agonist-1 activation by RR (Supplementary Fig. S9), the pore radius there was reduced as compared to that in mTRPV4_apo_ (Fig. 1v).

Like GSK101, we observed that Agonist-1 also induced upward movements in the S4-S5 linker and S5 (Fig. 1w), leading to the expansion in the S6 bundle crossing (Fig. 1x). Furthermore, Agonist-1 caused all the transmembrane domains to move in the clockwise direction by about 19.8° as compared to the mTRPV4_apo_ (Fig. 1y), facilitating the opening of the channel.

In summary, we have identified the cytosol-facing cavity in the S1 to S4 bundle as a hot spot for GSK101 and Agonist-1 binding in TRPV4 (Supplementary Fig. S10). 4α-PDD, another TRPV4 agonist, has also been suggested to bind there in two studies deposited in bioRxiv^10,11^. Moreover, other agonists also bind to this pocket in TRPV3 (2-APB)^12^, TRPV6 (2-APB)^13^, and TRPM8 (icilin)^14^, respectively. Agonist binding leads to upward movements in the S4-S5 linker and S5, which further opens the S6 bundling crossing. The selectivity filter of mouse TRPV4 adopts an open conformation in the apo state, which is distinct from those in TRPV1 or TRPV2. Like a plug, RR enters the selectivity filter from the extracellular side to block ion permeation in both TRPV6 reported previously^8^ and here in TRPV4.

Furthermore, when point mutations in diseases^2,15^ are mapped onto TRPV4 structure, they are clustered in the ankyrin repeats domain, S5, and the ligand binding pocket for GSK101 and Agonist-1 (Supplementary Fig. S11a). For instance, the F592L mutation found in patients with dysplasia^15^ virtually abolished ligand activation by GSK101 at negative membrane potential (Supplementary Fig. S11b, c), as it locates inside the ligand binding pocket. Therefore, elucidating the structural basis of ligand activation and inhibition in mammalian TRPV4 will help understand disease mechanisms and guide drug discovery targeting this channel in the future.

## Supplementary information

Supplementary Information

## Data Availability

Structure coordinates and cryo-EM density maps have been deposited in the Protein Data Bank under accession numbers 8J1D and EMD-35919 for mTRPV4_apo_, 8J1F and EMD- 35921 for mTRPV4_GSK101_, 8J1B and EMD-35918 for mTRPV4_GSK101_RR_, 8J1H and EMD-35922 for mTRPV4_Agonist1_RR_, and 8JKM and EMD-36373 for mTRPV4_RN1747_.
