# Supplementary material for: Structural basis of ligand activation and inhibition in a mammalian TRPV4 ion channel
Source: Cell Discov. 2023 Jul 10;9:70. doi: 10.1038/s41421-023-00579-3 (PMC10333285; doi:10.1038/s41421-023-00579-3)
Supplement: Supplementary file 1 — Supplementary Information [file 41421_2023_579_MOESM1_ESM.pdf]

Supplementary information

Materials and Methods

Protein expression and purification

$mTRPV4_{apo}$, $mTRPV4_{GSK}$, $mTRPV4_{GSK\_RR}$, $mTRPV4_{Agonist1\_RR}$ and $mTRPV4_{RN1747}$ were cloned, expressed, and purified following similar procedures. The *mouse TRPV4* gene fragment encoding residues 137-801 containing an N-terminal Flag and Strep tag II was cloned into a pEG-BM vector. This truncated TRPV4 exhibited similar ligand activation by GSK101 as the wildtype (full length) channel (Fig. S12), so we used this truncated TRPV4 for protein expression. All TRPV4s were heterologously expressed in Human Embryonic Kidney (HEK) 293F $GnTI^-$ suspension cells (Life Technologies) using the BacMam system (Thermo Fisher Scientific). The P2 baculovirus generated in Sf9 cells (Life Technologies) following standard protocol was used to transfect HEK 293F $GnTI^-$ cells at a ratio of 1:10 (virus: HEK293F, v/v) when the cell density is around 4×10^6 cells/mL. 10 mM sodium butyrate was added 12 h post-transduction, and cells were maintained at 30 °C to enhance protein expression. Cells were harvested by centrifugation at 4,000 rpm, and then flash-frozen in liquid nitrogen and stored at -80 °C.

The cell pellet was resuspended in lysis buffer A (20 mM Tris pH 8.0, 150 mM NaCl) supplemented with a protease inhibitor cocktail (2 μg/ml DNase I, 0.5 μg/ml pepstatin, 2 μg/ml leupeptin, 1 μg/ml aprotinin, and 100 μM phenylmethylsulfonyl fluoride) and homogenized by sonication on ice. mTRPV4 protein was extracted with 1% (w:v) n-Dodecyl-β-D-Maltopyranoside (DDM, Anatrace) by gentle agitation for 3 hours at 4 °C. After extraction, the supernatant was collected following a 1-hour ultra-centrifugation at 20000 rpm and then incubated gently with 0.5 mL Strep-Tactin Sepharose resin (IBA) at 4 °C. After 1 hour, the resin was collected on a gravity column (Bio-Rad) and washed in buffer B (buffer A + 0.03% GDN) for 20 column volumes. The detergent was then changed to 0.03% glyco-diosgenin (GDN, Anatrace), and the protein sample was eluted with 10 mM desthiobiotin (Sigma) in buffer C (buffer B + 10 mM desthiobiotin). The protein sample was further purified by size-exclusion chromatography on a Superose 6 10/300 GL column (GE Healthcare) pre-equilibrated with buffer B (20 mM Tris pH 8.0, 150 mM NaCl, 0.03% GDN). The peak fraction of TRPV4 was collected and concentrated in a 100-kDa concentrator (Amicon Ultra, Millipore Sigma) to appropriate concentrations (5 mg/ml) for cryo-EM analysis.

Reconstitution of TRPV4 into nanodisc

Membrane scaffold protein (MSP1E3D1) was expressed and purified from *Escherichia coli*. 1-palmitoyl-2-oleoyl-sn-glycero-3-phosphocholine (POPC) solubilized in chloroform (Avanti) was dried and dissolved in 3% DDM buffer thoroughly at a concentration of 15 mM. After eluted, TRPV4 was concentrated down to approximately 1 ml and mixed with lipid and MSP1E3D1 at a molar ratio of 1:10:2 (TRPV4: POPC: MSP1E3D1). The mixture was equilibrated at 4 °C for one hour and then added Bio-beads SM2 (Bio-Rad) two times within 16 h to gradually remove detergents. The reconstituted protein was separated from empty nanodisc by size-exclusion chromatography on a Superose 6 10/300 GL column pre-equilibrated with buffer D (20 mM Tris pH 8.0, 150 mM NaCl). The peak fractions were

collected and concentrated to 5 mg/ml for cryo-EM analysis.

Cryo-EM sample preparation and data acquisition

The cryo-EM data of all TRPV4 structures in either detergent ($mTRPV4_{apo}$, $mTRPV4_{GSK}$, $mTRPV4_{RN1747}$) or nanodisc ($mTRPV4_{GSK\_RR}$ and $mTRPV4_{Agonist1\_RR}$) was collected at Center of Cryo-Electron Microscopy at Zhejiang University. For the sample of $mTRPV4_{GSK}$, GSK101 was added to the sample of $mTRPV4_{GSK}$ to a final concentration of 100 μM. For the sample of $mTRPV4_{GSK\_RR}$, RR was added to the sample of $TRPV4_{GSK}$ to a final concentration of 500 μM. For the sample of $mTRPV4_{Agonist1\_RR}$, Agonist1 was added to the sample of $mTRPV4_{apo}$ to a final concentration of 100 μM, RR was added to the sample to a final concentration of 500 μM. For the sample of $mTRPV4_{RN1747}$, RN1747 was added to the sample of $mTRPV4_{RN1747}$ to a final concentration of 100 μM. The cryo-EM grids were prepared by applying 3 μl of TRPV4 protein sample to a glow-discharged holey carbon grid (Quantifoil Cu R1.2/1.3, 300 mesh) and blotted for 4.5 seconds under 100% humidity at 4 °C before being plunged into liquid ethane using a Mark IV Vitrobot (FEI).

Micrographs of $mTRPV4_{apo}$, $mTRPV4_{GSK}$, $mTRPV4_{GSK\_RR}$, $mTRPV4_{Agonist1\_RR}$ and $mTRPV4_{RN1747}$ were acquired on a Titan Krios microscope (FEI) operated at 300 kV equipped with the Selectris energy filter and Falcon4 detector. EPU software was used for automated data collection following standard procedures. A calibrated magnification of ×140,000 was used for imaging, yielding a pixel size of 0.93 Å on images. The defocus range was set from –0.8 to –1.6 μm. Each micrograph was dose-fractionated to 40 frames under a dose rate of 7.49 e^-/pixel/s, with a total exposure time of 6 s, resulting in a total dose of about 52 e^-/Å^2.

Image processing

For all datasets, motion correction, CTF estimation, particle picking, particle extraction and particle polishing were performed in Relion(version-4.0). Other data processes were performed using cryoSPARC (version-3.3.2).

For $mTRPV4_{apo}$, 2,812,534 particles were automatically picked from 3,001 images and after three rounds heterogeneous 3D refinement, 91,328 good-quality particles were finally selected for non-uniform 3D refinement with C4 symmetry and generated reconstruction map at 3.6 Å resolution with a B-factor of 106 Å^2.

For $mTRPV4_{GSK}$, 2,392,714 particles were initially extracted from 3,032 images. After 2D classification, ab initio reconstruction, and two rounds heterogeneous 3D refinement, 234,637 good-quality particles were selected for non-uniform 3D refinement with C4 symmetry and generated reconstruction map at 3.6 Å resolution with a B-factor of 135 Å^2.

For $mTRPV4_{GSK\_RR}$, 2,296,249 particles automatically picked from 2,985 images were performed three rounds heterogeneous 3D refinement. Finally 133,030 good-quality particles were selected for non-uniform 3D refinement with C4 symmetry and generated reconstruction map at 3.7 Å resolution with a B-factor of 111 Å^2.

For $mTRPV4_{Agonist1\_RR}$, 1,527,550 particles were automatically picked from 2,403 images and subjected to seven rounds heterogeneous 3D refinement. Finally 162,371 good-quality particles were selected for non-uniform 3D refinement with C4 symmetry and generated reconstruction map at 3.9 Å resolution with a B-factor of 143 Å^2.

For $mTRPV4_{RN1747}$, 622,583 particles were automatically picked from 2,001 images and subjected to heterogeneous 3D refinement. To generate the templates for template-based particle picking, after the first round of 2D classification, the 2D class of TRPV4 side-view was selected and used as the template. We have used the tool “Remove Duplicate Particles” in the Cryosparc software to deal with the duplication of the combined particles. Finally 74,344 good-quality particles were selected for non-uniform 3D refinement with C4 symmetry and generated reconstruction map at 3.8 Å resolution with a B-factor of 150 $Å^2$.

Model building, refinement, and validation

De novo atomic model building was performed in Coot based on the 3.59 Å resolution density map of $mTRPV4_{apo}$. The $mTRPV4_{apo}$ atomic model building using the hTRPV4 (PDB: 7AA5) model as a starting template. The amino acid assignment was achieved based on the clearly defined density for bulky residues (Phe, Trp, Tyr, and Arg). Models were refined against summed maps using real-space refinement in PHENIX[1], with secondary structure restraints and non-crystallography symmetry applied. The initial EM density map allowed us to construct an $mTRPV4_{apo}$ model containing residues 148–638 and 649–786. The model of all other TRPV4 structure was built using the model of $mTRPV4_{apo}$ as a template. The Van der Waals radii of the pore were calculated using HOLE[2]. All figures were prepared in PyMoL[3], Chimera[4], or Coot[5].

Chemicals

GSK1016790A, TRPV4 agonist-1 free base and RN-1747 were purchased from MCE. Ruthenium Red was purchased from Shanghai yuanye.

Cell culture for electrophysiology

HEK293T cells are cultured using DMEM medium contained 10% FBS in a cell incubator with 5% CO_2 at 37 °C. Plasmids of mTRPV4 are labeled with YFP as indicators for subsequent electrophysiological recordings. cDNA constructs of these ion channels were transiently transfected with Lipofectamine 2000 (Invitrogen, Carlsbad, CA). And one or two days after transient transfection, electrophysiological recordings were performed.

Site mutations of mTRPV4 were generated by Fast Mutagenesis Kit (Vazyme). Primers used to generate point mutations are summarized in Supplementary information, Table S5. All mutations were confirmed by sequencing.

Electrophysiology

Patch-clamp recordings were carried out with a HEKA EPC10 amplifier controlled by PatchMaster software (HEKA). Patch pipettes were prepared from borosilicate glass and fire-polished to resistance of 3~8 MΩ by P-97 puller for whole-cell recordings. Both bath solution and pipette solution contained 130 mM NaCl, 10 mM glucose, 0.2 mM EDTA, and 3 mM Hepes and were adjusted to pH 7.2 ~ 7.4 with NaOH for whole-cell recordings. Whole-cell recordings were performed at ±80 mV. Current was sampled at 10 kHz and filtered at 2.9 kHz. All recordings were performed at room temperature (25°C) with the maximum variation of 1 °C.

A gravity-driven system (RSC-200, Bio-Logic) with freely rotated perfusion tubes was

used for the perfusion of ligands. Bath and ligand solution were delivered through separate tubes to minimize the mixing of solutions. Patch pipette holding cells was placed in front of the perfusion tube outlet for perfusion.

Molecular dynamic simulation

Starting from $mTRPV4_{GSK101}$ and $mTRPV4_{Agonist1\_RR}$ structures, two MD simulation systems were set up with the proteins embedded in 1-palmitoyl- 2-oleoyl-sn-glycero-3-phosphocholine (POPC) bilayer with CHARMM-GUI[6,7]. The systems were solvated in 0.15 M NaCl solution with a total of ~ 5480,000 atoms in a cubic box of 150Å×150Å×140Å. Simulations were performed using OpenMM with the CHARMM36 force field[8,9] and TIP3P water model[10]. The system was equilibrated in six steps with gradually decreasing restraining force constants on the protein. Gradually reduced constraints were applied during the equilibration. Three repeats of 200 ns unrestrained atomistic simulations were then performed for each system, respectively. The Particle Mesh Ewald (PME) method[11] was used to model the long-range electrostatics (< 1 nm). Temperature coupling was done with V-rescale thermostat[12] at 293 K. The Parrinello-Rahman barostat[13] with a reference pressure of 1 bar and a compressibility of 4.5e-5 bar was applied for pressure control. Covalent bonds are constrained to their equilibrium length by the LINCS algorithm[14] to allow a 2 fs integration steps for production runs. The visualization and analysis were performed using VMD[4].

Data analysis

Data from whole-cell recordings were analyzed in Igor Pro version 6.11 (WaveMatrix). Hill Equation was used to fit the concentration-response curves for the calculation of EC50 and IC50.

References

1 Adams, P. D. *et al.* PHENIX: a comprehensive Python-based system for macromolecular structure solution. *Acta Crystallogr D Biol Crystallogr* **66**, 213-221, doi:10.1107/S0907444909052925 (2010).

2 Smart, O. S., Goodfellow, J. M. & Wallace, B. A. The pore dimensions of gramicidin A. *Biophys J* **65**, 2455-2460, doi:10.1016/S0006-3495(93)81293-1 (1993).

3 Schrodinger, LLC. *The PyMOL Molecular Graphics System, Version 1.8* (2015).

4 Pettersen, E. F. *et al.* UCSF Chimera--a visualization system for exploratory research and analysis. *Journal of computational chemistry* **25**, 1605-1612, doi:10.1002/jcc.20084 (2004).

5 Emsley, P., Lohkamp, B., Scott, W. G. & Cowtan, K. Features and development of Coot. *Acta crystallographica. Section D, Biological crystallography* **66**, 486-501, doi:10.1107/S0907444910007493 (2010).

6 Jo, S., Kim, T., Iyer, V. G. & Im, W. CHARMM-GUI: a web-based graphical user interface for CHARMM. *Journal of computational chemistry* **29**, 1859-1865, doi:10.1002/jcc.20945 (2008).

7 Lee, J. *et al.* CHARMM-GUI Input Generator for NAMD, GROMACS, AMBER, OpenMM, and CHARMM/OpenMM Simulations Using the CHARMM36 Additive Force Field. *Journal of Chemical Theory and Computation* **12**, 405-413, doi:10.1021/acs.jctc.5b00935 (2016).

8 Huang, J. & MacKerell, A. D., Jr. CHARMM36 all-atom additive protein force field:

validation based on comparison to NMR data. *J Comput Chem* **34**, 2135-2145, doi:10.1002/jcc.23354 (2013).

9 Klauda, J. B. *et al.* Update of the CHARMM All-Atom Additive Force Field for Lipids: Validation on Six Lipid Types. *The Journal of Physical Chemistry B* **114**, 7830-7843, doi:10.1021/jp101759q (2010).

10 Jorgensen, W. L., Chandrasekhar, J., Madura, J. D., Impey, R. W. & Klein, M. L. Comparison of simple potential functions for simulating liquid water. *The Journal of chemical physics* **79**, 926-935, doi:10.1063/1.445869 (1983).

11 Essmann, U. *et al.* A smooth particle mesh Ewald method. *The Journal of chemical physics* **103**, 8577-8593, doi:10.1063/1.470117 (1995).

12 Bussi, G., Donadio, D. & Parrinello, M. Canonical sampling through velocity rescaling. *The Journal of chemical physics* **126**, 014101, doi:10.1063/1.2408420 (2007).

13 Parrinello, M. & Rahman, A. Polymorphic transitions in single crystals: A new molecular dynamics method. *Journal of Applied Physics* **52**, 7182-7190, doi:10.1063/1.328693 (1981).

14 Hess, B., Bekker, H., Berendsen, H. J. C. & Fraaije, J. G. E. M. LINCS: A linear constraint solver for molecular simulations. *Journal of Computational Chemistry* **18**, 1463-1472, doi:https://doi.org/10.1002/(SICI)1096-987X(199709)18:12<1463::AID-JCC4>3.0.CO;2-H (1997).

Supplementary information, Figures

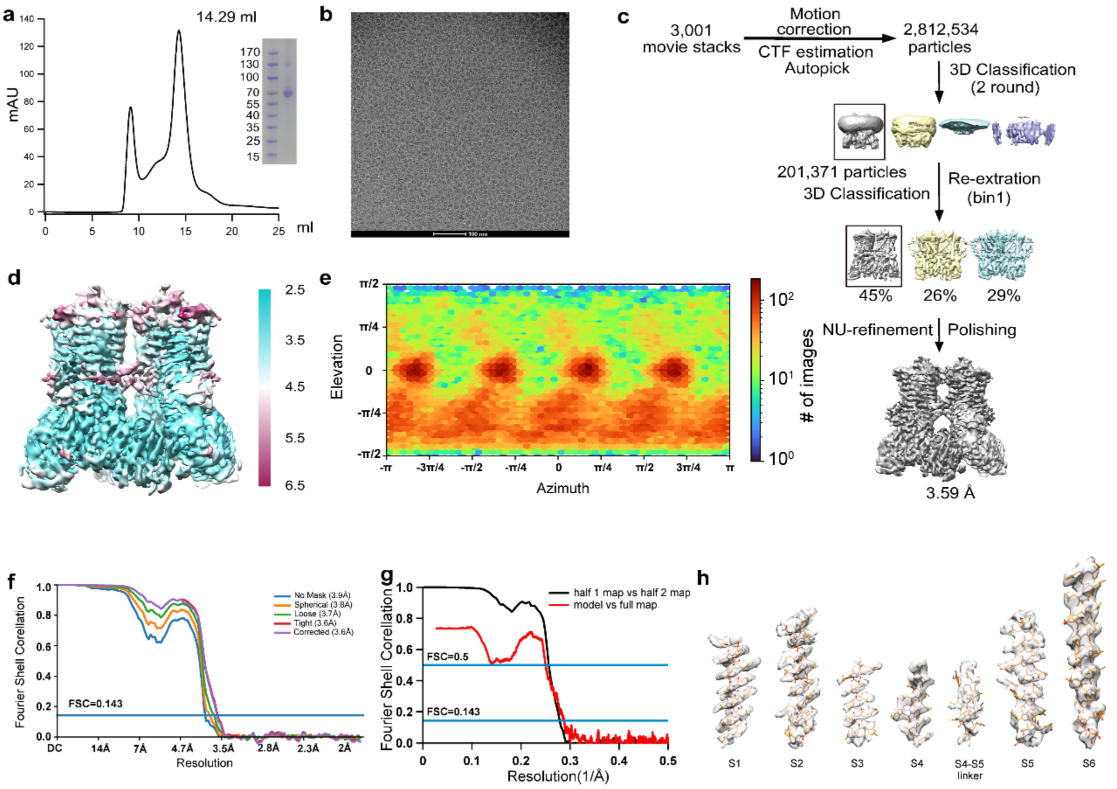

Figure S1. **Structure determination of mTRPV4apo. a** Size-exclusion chromatography of mTRPV4apo on Superose 6 (GE Healthcare) and SDS-PAGE analysis of the final sample. **b** Representative cryo-EM micrograph of mTRPV4apo. **c** Flowchart of image processing for mTRPV4apo particles. **d** The density map of mTRPV4apo colored by local resolution (Å). **e** Distribution of orientations over azimuth and elevation angles for particles included in the calculation of the final map. **f** The Gold standard Fourier Shell Correlation (FSC) curve of the final 3D reconstruction of mTRPV4apo with different masking, as calculated in cryoSPARC. **g** The FSC curves of the final 3D reconstruction of mTRPV4apo, and the FSC curve for cross-validation between the map and the model of mTRPV4apo. **h** Sample maps of six transmembrane helices (TMs) in mTRPV4apo.

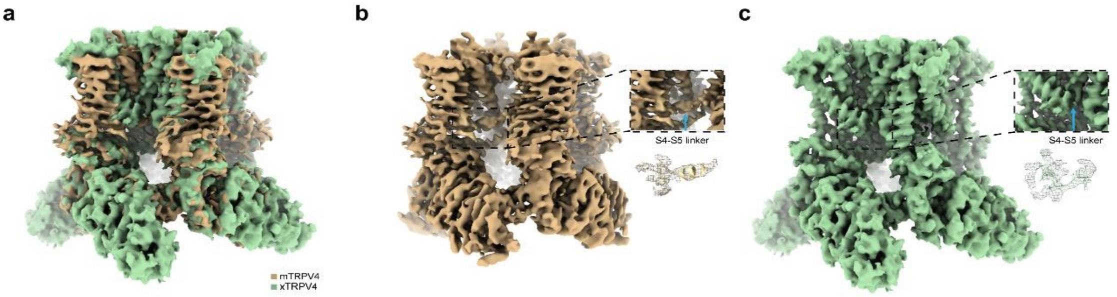

Figure S2. 3D reconstruction of mTRPV4 compared to xTRPV4. a 3D reconstruction of mTRPV4 compared to xTRPV4 in the apo state. **b** 3D reconstruction of mTRPV4 in the apo state. The electron density of S4-S5 linker of mTRPV4 is shown in an enlarged view. **c** 3D reconstruction of xTRPV4 in the apo state. The electron density of S4-S5 linker of xTRPV4 is shown in an enlarged view.

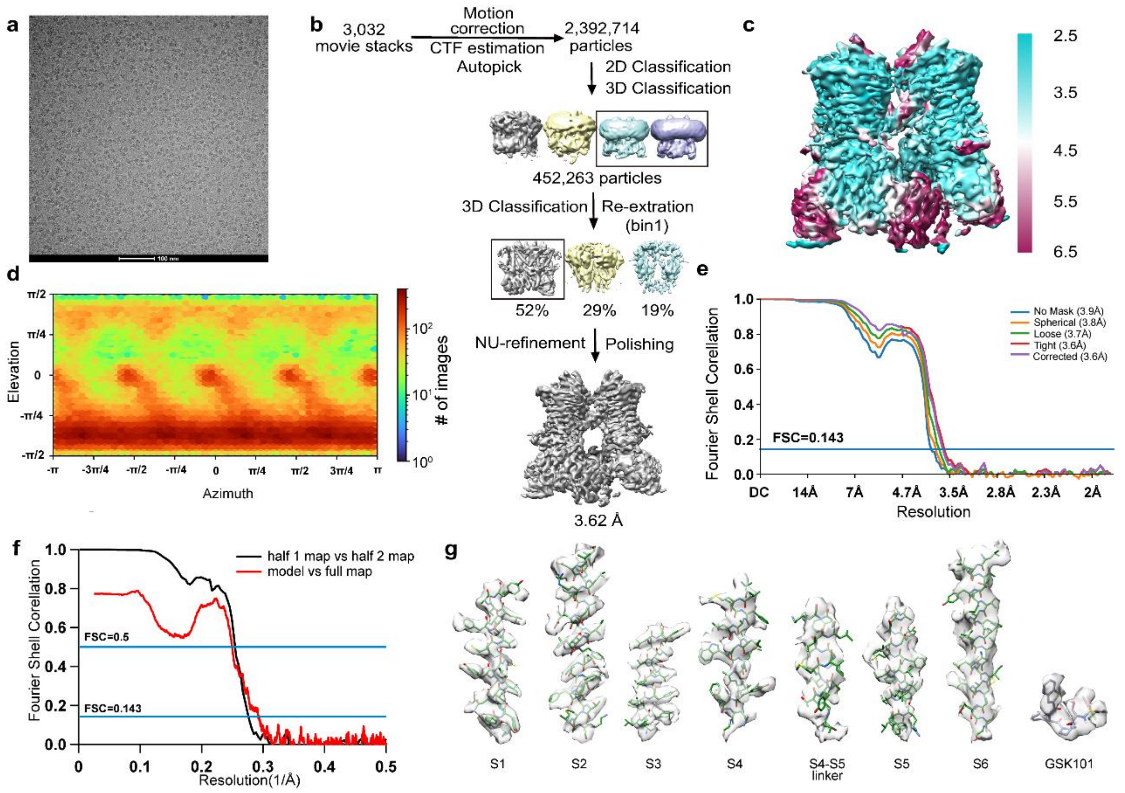

Figure S3. **Structure determination of mTRPV4$_{GSK101}$. a** Representative cryo-EM micrograph of mTRPV4$_{GSK101}$. **b** Flowchart of image processing for mTRPV4$_{GSK101}$ particles. **c** The density map of mTRPV4$_{GSK101}$ colored by local resolution (Å). **d** Distribution of orientations over azimuth and elevation angles for particles included in the calculation of the final map. **e** The Gold standard Fourier Shell Correlation (FSC) curve of the final 3D reconstruction of mTRPV4$_{GSK101}$ with different masking, as calculated in cryoSPARC. **f** The FSC curves of the final 3D reconstruction of mTRPV4$_{GSK101}$, and the FSC curve for cross-validation between the map and the model of mTRPV4$_{GSK101}$. **g** Sample maps of six transmembrane helices (TMs) in mTRPV4$_{GSK101}$. The density corresponding to GSK101 is also shown.

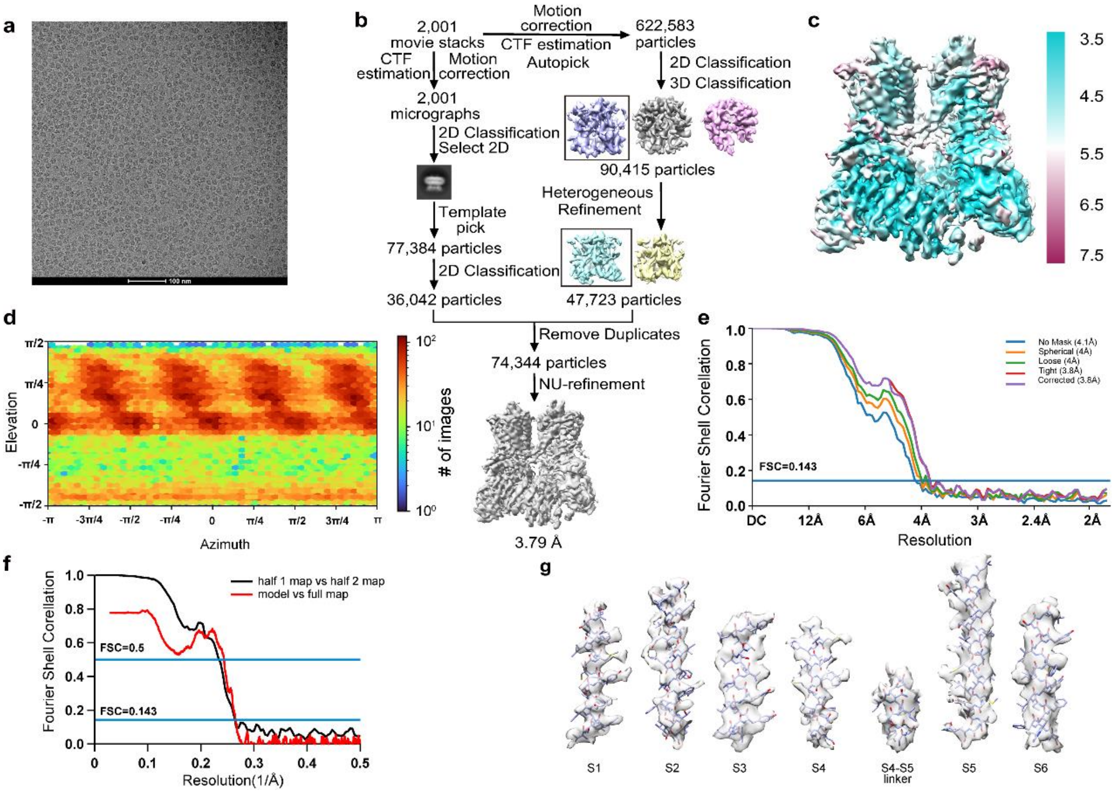

Figure S4. Structure determination of mTRPV4$_{RN1747}$. a Representative cryo-EM micrograph of mTRPV4$_{RN1747}$. **b** Flowchart of image processing for mTRPV4$_{RN1747}$ particles. **c** The density map of mTRPV4$_{RN1747}$ colored by local resolution (Å). **d** Distribution of orientations over azimuth and elevation angles for particles included in the calculation of the final map. **e** The Gold standard Fourier Shell Correlation (FSC) curve of the final 3D reconstruction of mTRPV4$_{RN1747}$ with different masking, as calculated in cryoSPARC. **f** The FSC curves of the final 3D reconstruction of mTRPV4$_{RN1747}$, and the FSC curve for cross-validation between the map and the model of mTRPV4$_{RN1747}$. **g** Sample maps of six transmembrane helices (TMs) in mTRPV4$_{RN1747}$.

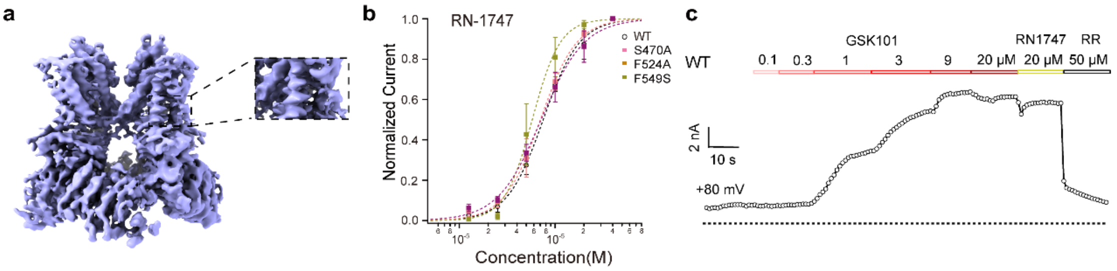

Figure S5. RN-1747 does not bind to the pocket of GSK101 or Agonist-1. a 3D reconstruction of mTRPV4$_{RN1747}$. RN-1747 is not observed in the binding site of GSK101 and Agonist-1, which is shown in an enlarged view. **b** Concentration-response curves of RN-1747 activation of WT and mutants from the membrane in whole-cell patch-clamp recordings (n = 3–4 cells). Data presented as mean ± s.e.m. **c** Representative current recordings of WT mTRPV4, GSK101-activated current is normalized by RN-1747-activated current.

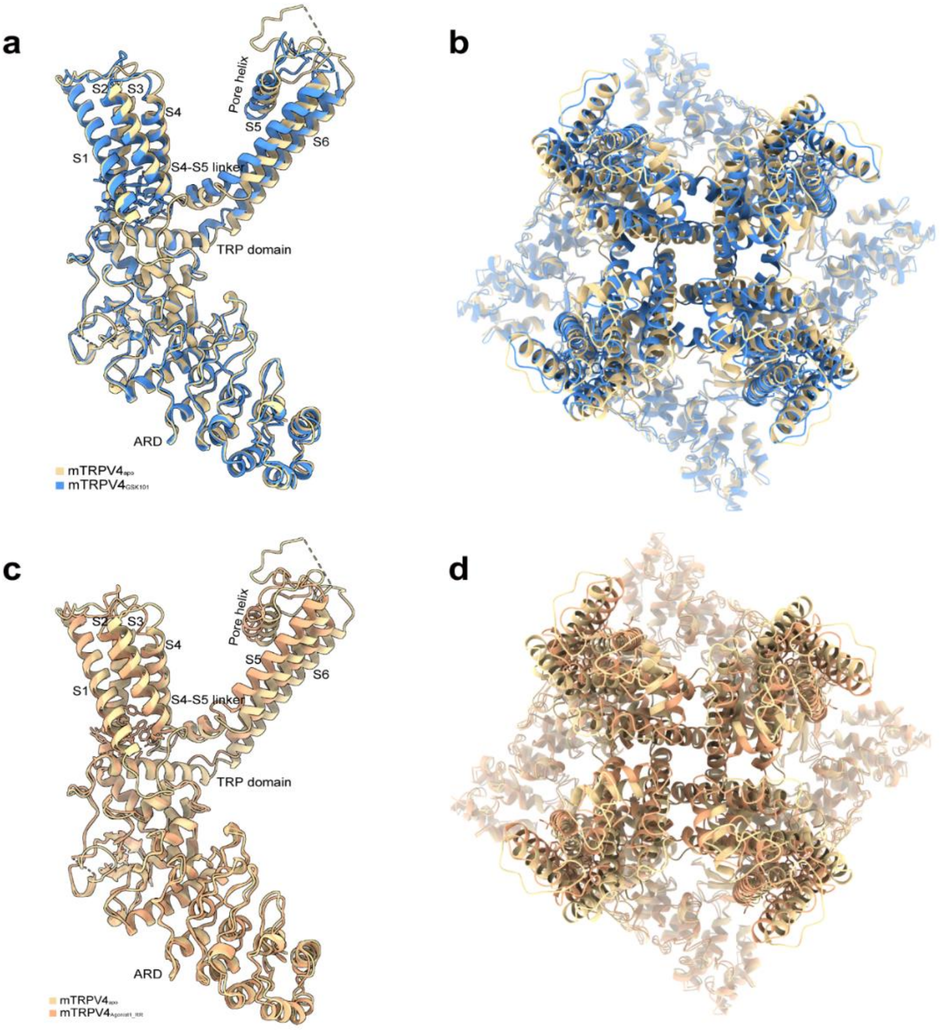

Figure S6. Conformational changes of mTRPV4$_{apo}$ compared to mTRPV4$_{GSK}$ and mTRPV4$_{Agonist1_RR}$. a Side view of the conformational changes of mTRPV4$_{GSK}$ compared with mTRPV4$_{apo}$. Only one subunit was shown for clarity. **b** Top view from the extracellular side of the conformational changes of mTRPV4$_{GSK}$ compared with mTRPV4$_{apo}$. **c** Side view of the conformational changes of mTRPV4$_{Agonist1_RR}$ compared with mTRPV4$_{apo}$. Only one subunit was shown for clarity. **d** Top view from the extracellular side of the conformational changes of mTRPV4$_{Agonist1_RR}$ compared with mTRPV4$_{apo}$.

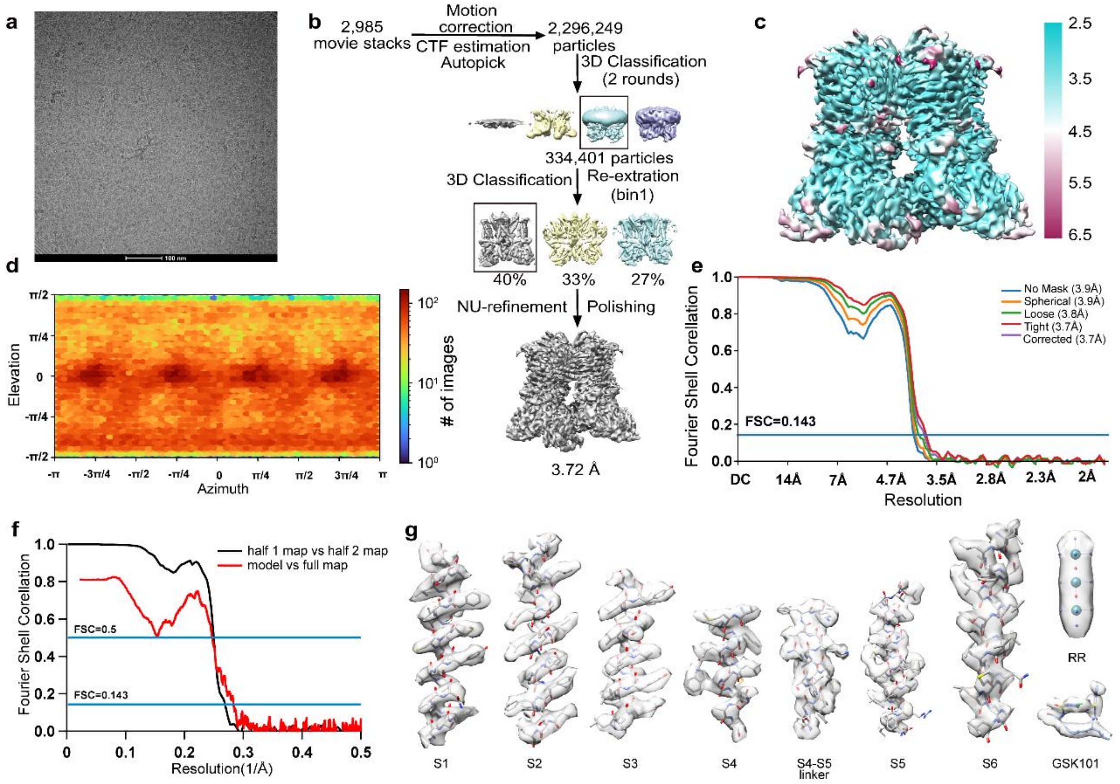

Figure S7. **Structure determination of mTRPV4$_{GSK101_RR}$.** **a** Representative cryo-EM micrograph of mTRPV4$_{GSK101_RR}$. **b** Flowchart of image processing for mTRPV4$_{GSK101_RR}$ particles. **c** The density map of mTRPV4$_{GSK101_RR}$ colored by local resolution (Å). **d** Distribution of orientations over azimuth and elevation angles for particles included in the calculation of the final map. **e** The Gold standard Fourier Shell Correlation (FSC) curve of the final 3D reconstruction of mTRPV4$_{GSK101_RR}$ with different masking, as calculated in cryoSPARC. **f** The FSC curves of the final 3D reconstruction of mTRPV4$_{GSK101_RR}$, and the FSC curve for cross-validation between the map and the model of mTRPV4$_{GSK101_RR}$. **g** Sample maps of six transmembrane helices (TMs) in mTRPV4$_{GSK101_RR}$. The density corresponding to GSK101 and Ruthenium Red is also shown.

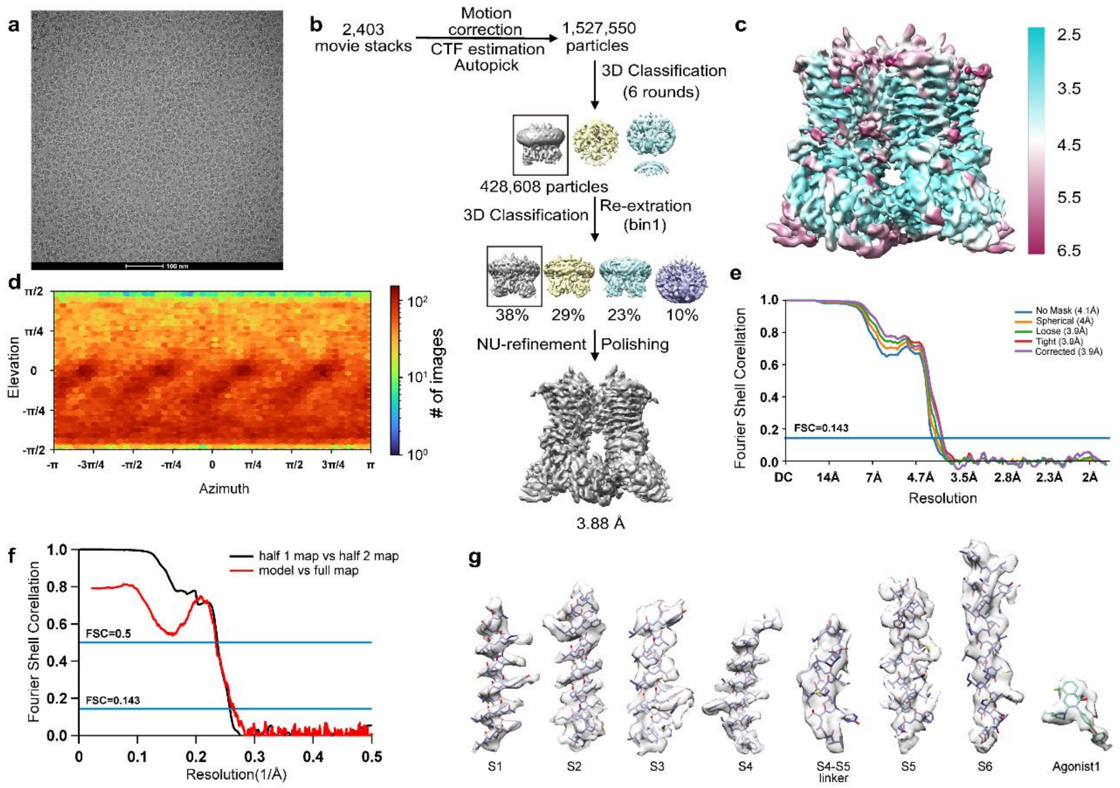

Figure S8. Structure determination of mTRPV4$_{\mathbf{Agonist1_RR}}$. a Representative cryo-EM micrograph of mTRPV4$_{Agonist1_RR}$. **b** Flowchart of image processing for mTRPV4$_{Agonist1_RR}$ particles. **c** The density map of mTRPV4$_{Agonist1_RR}$ colored by local resolution (Å). **d** Distribution of orientations over azimuth and elevation angles for particles included in the calculation of the final map. **e** The Gold standard Fourier Shell Correlation (FSC) curve of the final 3D reconstruction of mTRPV4$_{Agonist1_RR}$ with different masking, as calculated in cryoSPARC. **f** The FSC curves of the final 3D reconstruction of mTRPV4$_{Agonist1_RR}$, and the FSC curve for cross-validation between the map and the model of mTRPV4$_{Agonist1_RR}$. **g** Sample maps of six transmembrane helices (TMs) in mTRPV4$_{Agonist1_RR}$. The density corresponding to Agonist1 is also shown.

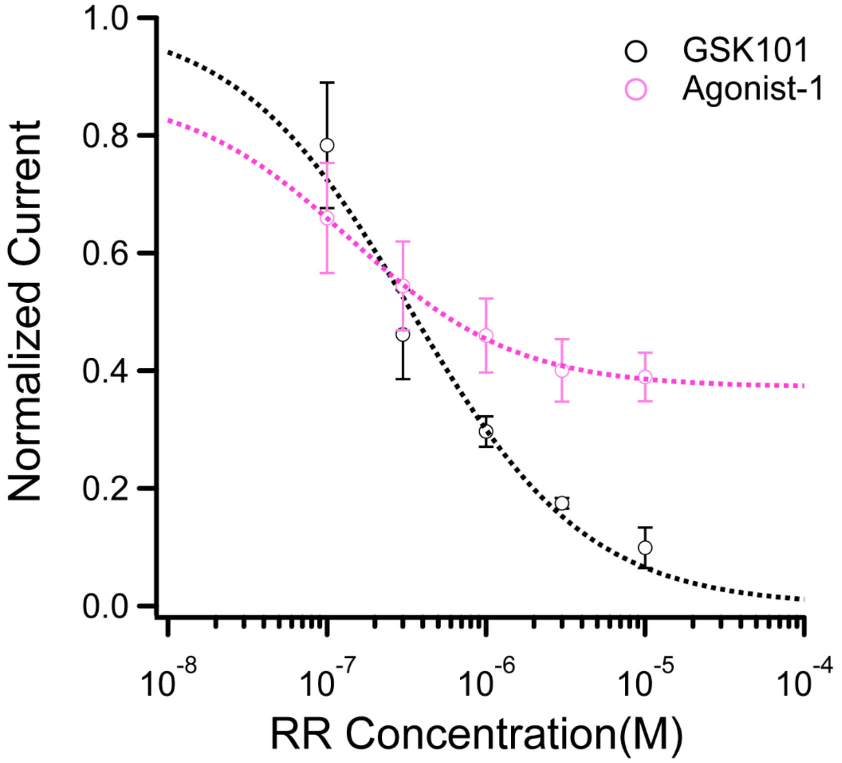

Figure S9. Electrophysiological characterization of Ruthenium Red inhibition in mTRPV4 channels activated by GSK101 and Agonist-1, respectively. Concentration-response curves of Ruthenium Red inhibition in WT mTRPV4 activated by GSK101 and Agonist-1 respectively in whole-cell patch-clamp recordings (n = 3–4 cells). Data are presented as mean ± s.e.m.

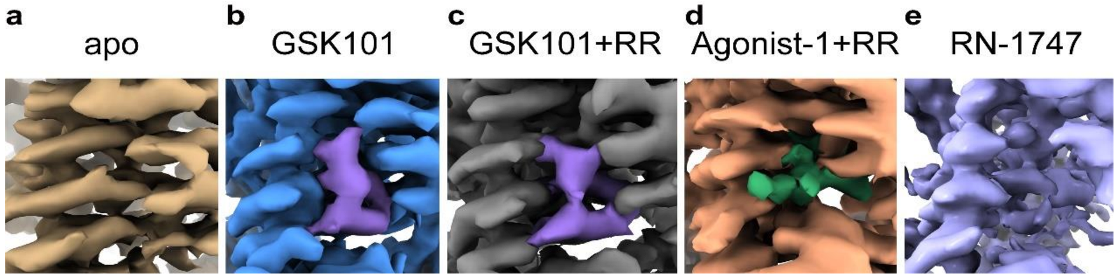

Figure S10. Close-up view at the ligand binding site of 3D reconstructions of mTRPV4. a Close-up view at the ligand binding site of 3D reconstructions of mTRPV4$_{apo}$. **b** Close-up view at the ligand binding site of 3D reconstructions of mTRPV4$_{GSK101}$. **c** Close-up view at the ligand binding site of 3D reconstructions of mTRPV4$_{GSK101_RR}$. **d** Close-up view at the ligand binding site of 3D reconstructions of mTRPV4$_{Agonist1_RR}$. **e** Close-up view at the ligand binding site of 3D reconstructions of mTRPV4$_{RN1747}$.

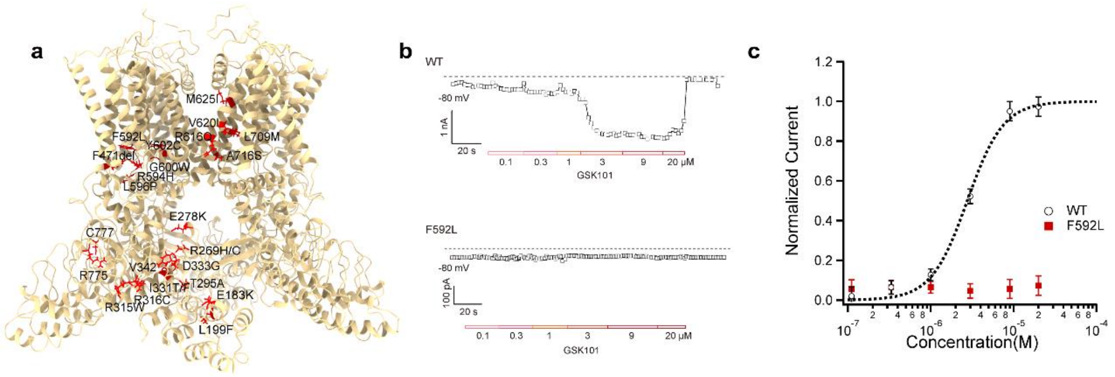

Figure S11. Structural mapping of TRPV4 disease mutations. a The locations of the mutations relative to the domains of $mTRPV4_{apo}$ for autosomal-dominant brachyolmia, spondylometaphyseal dysplasia, Kozlowski type, and metatropic dysplasia. **b** Representative current recordings of WT and mutant F592L, where GSK101 activation is abolished at -80 mV. **c** Concentration-response curves of GSK101 activation of WT and F592L from the membrane in whole-cell patch-clamp recordings (n = 3–4 cells). Data presented as mean ± s.e.m.

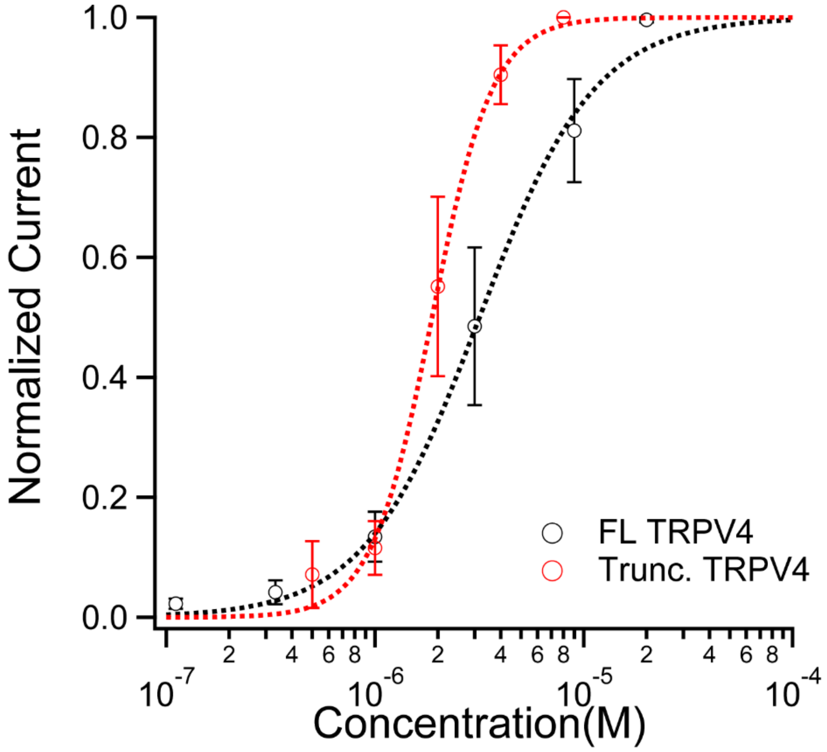

Figure S12. **Electrophysiological characterization of full-length and truncated mTRPV4 channels.** Concentration-response curves of GSK101 activation of full-length and truncated mTRPV4 channels from the membrane in whole-cell patch-clamp recordings (n = 5–8 cells). Data presented as mean ± s.e.m.

Supplementary information, Table S1

Cryo-EM data collection, refinement and validation statistics

	mTRPV4	mTRPV4	mTRPV4	mTRPV4	mTRPV4
	apo	GSK101	GSK101_RR	Agonist1_RR	RN1747
**Data collection and processing**	mTRPV4	mTRPV4	mTRPV4	mTRPV4	mTRPV4
	apo	GSK101	GSK101_RR	Agonist1_RR	RN1747
Magnification	49,310×	49,310×	49,310×	49,310×	49,310×
Voltage (kV)	300	300	300	300	300
Electron exposure ($e–/Å^2$)	~52	~52	~52	~52	~52
Defocus range (μm)	-0.8 to -1.6	-0.8 to -1.6	-0.8 to -1.6	-0.8 to -1.6	-0.8 to -1.6
Pixel size (Å)	0.93	0.93	0.93	0.93	0.93
Symmetry imposed	*C4*	*C4*	*C4*	*C4*	*C4*
Initial particle images (no.)	2,812,534	2,392,714	2,296,249	1,527,550	622,583
Final particle images (no.)	201,371	452,263	334,401	428,608	74,344
Map resolution (Å)	3.59	3.62	3.72	3.88	3.79
FSC threshold	0.143	0.143	0.143	0.143	0.143
**Refinement**					
Initial model used (PDB code)	hTRPV4 (7AA5)	$mTRPV4_{apo}$	$mTRPV4_{apo}$	$mTRPV4_{apo}$	$mTRPV4_{apo}$
Model resolution (Å)	3.4	3.4	3.5	3.8	3.8
FSC threshold	0.143	0.143	0.143	0.143	0.143
Map sharpening *B* factor ($Å^2$)	-106	-135	-111	-143	-150
Model composition					
Non-hydrogen atoms	20,200	19,124	19,412	19,144	18,924
Protein residues	2,516	2,516	2,516	2,356	2,344
Ligands	0	4	5	5	0
B factors ($Å^2$)					
Protein	26.71	101.65	75.39	118.84	120.25
Ligand		94.99	53.68	110.93	
R.m.s. deviations					
Bond lengths (Å)	0.003	0.004	0.005	0.005	0.004
Bond angles (°)	0.594	0.967	1.279	1.032	0.948
Validation					
MolProbity score	2.23	2.07	2.35	2.42	2.14
Clashscore	14.55	13.50	18.75	20.72	16.09
Poor rotamers (%)	0.09	0.00	0.19	0.19	0.00
Ramachandran plot					
Favored (%)	89.72	93.41	88.88	87.48	93.45
Allowed (%)	9.36	6.41	10.09	12.00	6.38
Disallowed (%)	0.92	0.17	1.02	0.52	0.17

Supplementary information, Table S2

EC50 values of GSK101 activation of TRPV4 and its mutants.

**Mutant**	**EC50（μM）**	**n**
TRPV4 WT	1.6 ± 0.2	5
TRPV4 WT(-80 mV)	2.8 ± 0.2	4
Trunc. TRPV4	1.9 ± 0.1	5
S470A	6.4 ± 0.2	3
F524A	6.6 ± 0.1	3
T527Q	0.5 ± 0.1	5
F549S	3.5 ± 1.3	5

Supplementary information, Table S3

IC50 values of Ruthenium red inhibition of TRPV4 and its mutants.

**Mutant**	**EC50（μM）**	**n**	**Current activated by 2 μM GSK101**
TRPV4 WT	0.3 ± 0.1	3	3.2 ± 1.2
T677A	0.8 ± 0.1	4	2.3 ± 0.4
I678A	0.6 ± 0.1	3	3.5 ± 1.3
G679A	0.6 ± 0.1	3	3.6 ± 1.3
M680A	0.7 ± 0.2	3	1.8 ± 0.2
G681A	0.1 ± 0.1	3	3.5 ± 1.1
D682A	15.6 ± 3.2	3	5.3 ± 1.2

Supplementary information, Table S4

EC50 values of TRPV4 agonist-1 activation of TRPV4 and its mutants.

**Mutant**	**EC50（μM）**	**n**
TRPV4 WT	0.4 ± 0.1	4
S470A	2.1 ± 0.1	3
S470R	1.1 ± 0.1	4
N474A	1.6 ± 0.1	4
Y478A	1.0 ± 0.1	3
T527Q	1.8 ± 0.1	3
Y553F	2.1 ± 0.2	3
F592A	1.7 ± 0.1	3
F592S	1.0 ± 0.1	3
F592Q	1.0 ± 0.1	3

Supplementary information, Table S5
EC50 values of RN-1747 activation of TRPV4 and its mutants.

Mutant	EC50（μM）	n
TRPV4 WT	7.5 ± 0.2	3
S470A	7.0 ± 0.2	3
F524A	5.8 ± 0.1	3
F549S	7.1 ± 0.3	4

Supplementary information, Table S6
Primers used to generate TRPV4 mutations.

Name	Primer-Forward	Primer-Reverse
**S470A**	AAGTTTGGGGCTGTGGCCTTCTACATCAAC	GGCCACAGCCCCAAACTTACGCCACTTG
**S470F**	AAGTTTGGGGCTGTGTTCTTCTACATCAAC	GAACACAGCCCCAAACTTACGCCACTTG
**S470R**	AAGTTTGGGGCTGTGCGCTTCTACATCAAC	GCGCACAGCCCCAAACTTACGCCACTTGTC
**N474A**	GTGTCCTTCTACATCGCCGTGGTCTCCT	GGCGATGTAGAAGGACACAGCCCCAAAC
**N474S**	GTGTCCTTCTACATCAGCGTGGTCTCCT	GCTGATGTAGAAGGACACAGCCCCAAAC
**N474Q**	GTGTCCTTCTACATCGTTGTGGTCTCCT	AACGATGTAGAAGGACACAGCCCCAAAC
**N474F**	GTGTCCTTCTACATCTTCGTGGTCTCCT	GAAGATGTAGAAGGACACAGCCCCAAAC
**S477A**	TACATCAACGTGGTCGCCTATCTGTGTG	GGCGACCACGTTGATGTAGAAGGACACA
**S477Q**	TACATCAACGTGGTCCAATATCTGTGTG	TTGGACCACGTTGATGTAGAAGGACACA
**S477F**	TACATCAACGTGGTCTTCTATCTGTGTG	GAAGACCACGTTGATGTAGAAGGACACA
**Y478A**	ATCAACGTGGTCTCCGCTCTGTGTGCCA	AGCGGAGACCACGTTGATGTAGAAGGAC
**Y478S**	ATCAACGTGGTCTCCAGTCTGTGTGCCA	ACTGGAGACCACGTTGATGTAGAAGGAC
**Y478F**	ATCAACGTGGTCTCCTTTCTGTGTGCCA	AAAGGAGACCACGTTGATGTAGAAGGAC
**F524A**	TTCACAGGAGTCCTGGCCTTCTTTACCA	GGCCAGGACTCCTGTGAAGAGCGTGATG
**F524S**	TTCACAGGAGTCCTGTCTTTCTTTACCA	AGACAGGACTCCTGTGAAGAGCGTGATG
**T527A**	GTCCTGTTCTTCTTTGCCAGTATCAAAG	GGCAAAGAAGAACAGGACTCCTGTGAAG

**T527Q**	GTCCTGTTCTTCTTTCAAAGTATCAAAG	TTGAAAGAAGAACAGGACTCCTGTGAAG
**F549A**	TTCGTCGATGGCTCCGCCCAGTTACTCT	GGCGGAGCCATCGACGAAGAGAGAATTC
**F549S**	TTCGTCGATGGCTCCTCCCAGTTACTCT	GGAGGAGCCATCGACGAAGAGAGAATTC
**Q550A**	GTCGATGGCTCCTTCGCGTTACTCTACT	CGCGAAGGAGCCATCGACGAAGAGAGAA
**Q550S**	GTCGATGGCTCCTTCTCGTTACTCTACT	CGAGAAGGAGCCATCGACGAAGAGAGAA
**Q550N**	GTCGATGGCTCCTTCAACTTACTCTACT	GTTGAAGGAGCCATCGACGAAGAGAGAA
**Q550F**	GTCGATGGCTCCTTCTTCTTACTCTACT	GAAGGAGCCATCGACGAAGAGAGAATTC
**Y553A**	TCCTTCCAGTTACTCGCCTTCATCTACT	GGCGAGTAACTGGAAGGAGCCATCGACG
**Y553S**	TCCTTCCAGTTACTCTCCTTCATCTACT	GGAGAGTAACTGGAAGGAGCCATCGACG
**Y553F**	TCCTTCCAGTTACTCTTCTTCATCTACT	GAAGAGTAACTGGAAGGAGCCATCGACG
**Y591A**	TGGATGAATGCGCTGGCCTTCACGCGCG	GGCCAGCGCATTCATCCAGCCCAGGACC
**Y591S**	TGGATGAATGCGCTGTCCTTCACGCGCG	GGACAGCGCATTCATCCAGCCCAGGACC
**Y591F**	TGGATGAATGCGCTGTTCTTCACGCGCG	GAACAGCGCATTCATCCAGCCCAGGACC
**F592A**	AATGCGCTGTACGCCACGCGCGGGTTGAAGCTG	GTGGCGTACAGCGCATTCATCCAGCCCAGGAC
**F592S**	AATGCGCTGTACTCCACGCGCGGGTTGAAGCTG	GTGGAGTACAGCGCATTCATCCAGCCCAGGAC
**F592Q**	AATGCGCTGTACCAGACGCGCGGGTTGAAGCTG	GTCTGGTACAGCGCATTCATCCAGCCCAGGAC
**T677A**	GACCTCTTCAAGCTCGCCATCGGCATGG	GGCGAGCTTGAAGAGGTCCAGGAGGAAG
**I678A**	CTCTTCAAGCTCACCGCCGGCATGGGAG	GGCGGTGAGCTTGAAGAGGTCCAGGAGG
**G679A**	TTCAAGCTCACCATCGCCATGGGAGACC	GGCGATGGTGAGCTTGAAGAGGTCCAGG
**M680A**	AAGCTCACCATCGGCGCGGGAGACCTGG	CGCGCCGATGGTGAGCTTGAAGAGGTCC
**G681A**	CTCACCATCGGCATGGCAGACCTGGAGA	TGCCATGCCGATGGTGAGCTTGAAGAGG
**D682A**	ACCATCGGCATGGGAGCCCTGGAGATGC	GGCTCCCATGCCGATGGTGAGCTTGAAG

**D743A**	GCCACCACCATCCTGGCCATCGAGCGTT	GGCCAGGATGGTGGTGGCCCACTGCAAC
**D743E**	GCCACCACCATCCTGGAAATCGAGCGTT	TTCCAGGATGGTGGTGGCCCACTGCAAC
**D743R**	GCCACCACCATCCTGCGCATCGAGCGTT	GCGCAGGATGGTGGTGGCCCACTGCAAC
**I744A**	ACCACCATCCTGGACGCCGAGCGTTCCT	GGCGTCCAGGATGGTGGTGGCCCACTGC
**I744F**	ACCACCATCCTGGACTTCGAGCGTTCCT	GAAGTCCAGGATGGTGGTGGCCCACTGC
**S747A**	CTGGACATCGAGCGTGCCTTCCCTGTGT	GGCACGCTCGATGTCCAGGATGGTGGTG
**S747R**	CTGGACATCGAGCGTCGCTTCCCTGTGT	GCGACGCTCGATGTCCAGGATGGTGGTG
**S747F**	CTGGACATCGAGCGTTTCTTCCCTGTGT	GAAACGCTCGATGTCCAGGATGGTGGTG
